# Salivary proline-rich protein may reduce tannin-iron chelation: a systematic narrative review

**DOI:** 10.1186/s12986-017-0197-z

**Published:** 2017-07-24

**Authors:** Nicole M. Delimont, Sara K. Rosenkranz, Mark D. Haub, Brian L. Lindshield

**Affiliations:** Department of Food, Nutrition, Dietetics and Health, Kansas State University, 1324 Lovers Lane, 208 Justin Hall, Manhattan, KS, USA

**Keywords:** Iron bioavailability, Antinutritional factors, Tannin, Proanthocyanidins, Salivary proline-rich proteins

## Abstract

**Background:**

Tannins are often cited for antinutritional effects, including chelation of non-heme iron. Despite this, studies exploring non-heme iron bioavailability inhibition with long-term consumption have reported mixed results. Salivary proline-rich proteins (PRPs) may mediate tannin-antinutritional effects on non-heme iron bioavailability.

**Aim:**

To review evidence regarding biochemical binding mechanisms and affinity states between PRPs and tannins, as well as effects of PRPs on non-heme iron bioavailability with tannin consumption in vivo.

**Methods:**

Narrative systematic review and meta-analysis. Common themes in biochemical modeling and affinity studies were collated for summary and synthesis; data were extracted from in vivo experiments for meta-analysis.

**Results:**

Thirty-two studies were included in analysis. Common themes that positively influenced tannin-PRP binding included specificity of tannin-PRP binding, PRP and tannin stereochemistry. Hydrolyzable tannins have different affinities than condensed tannins when binding to PRPs. In vivo, hepatic iron stores and non-heme iron absorption are not significantly affected by tannin consumption (*d* = −0.64-1.84; −2.7-0.13 respectively), and PRP expression may increase non-heme iron bioavailability with tannin consumption.

**Conclusions:**

In vitro modeling suggests that tannins favor PRP binding over iron chelation throughout digestion. Hydrolyzable tannins are not representative of tannin impact on non-heme iron bioavailability in food tannins because of their unique structural properties and PRP affinities. With tannin consumption, PRP production is increased, and may be an initial line of defense against tannin-non-heme iron chelation in vivo*.* More research is needed to compare competitive binding of tannin-PRP to tannin-non-heme iron complexes, and elucidate PRPs’ role in adaption to non-heme iron bioavailability in vivo.

**Electronic supplementary material:**

The online version of this article (doi:10.1186/s12986-017-0197-z) contains supplementary material, which is available to authorized users.

## Background

### Non-heme iron-tannin binding

**Fig. 1:**
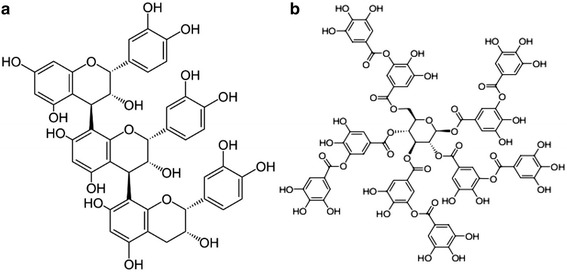
Condensed tannin (**a**). Tannic acid (**b**) [28]

Tannins are defensive metabolites classified as either hydrolyzable or condensed [1], that protect plants from insects, animal predators, and mold [2]. Hydrolyzable tannins are esters of polyols with phenolic acids (generally gallic acid) that are readily hydrolyzed by acidic or basic conditions [1], and are “virtually absent from the diet” [3] (Fig. 1a). Condensed tannins, also known as proanthocyanidins, are more commonly consumed in sorghum, wine, tea, dark chocolate and berries [3], and are comprised of oligomers and polymers of flavan-3-ols linked by carbon bonds [4] that are difficult to hydrolyze [1] (Fig. 1b).

While tannin-rich foods may confer potential antioxidant [5], cancer fighting [6], and cardiovascular [7] health benefits, a potential drawback to their consumption is that they inhibit non-heme iron bioavailability [8–20]. However, the evidence supporting this antinturient function have mostly been single meal studies utilizing hydrolyzable tannic acid or oligomeric epigallocatechin gallate found in tea. Studies that have explored long-term consumption of tannins [21, 22] and other antinutritional factors [23, 24], as well as epidemiological studies [25–27] have questioned whether potent tannin-non-heme iron inhibition is sustained over time.

Traditionally, protein-tannin binding has been cited as a major contributor to the antinutritional effect of tannins outside of mineral metabolism. It is tannins’ predilection toward protein interaction that may mediate non-heme iron-chelation by salivary proline rich proteins (PRPs), which bind to tannins in lieu of non-heme iron to make the mineral more bioavailable. Tannins’ ability to interact with proteins varies widely, and it has been noted that protein characteristics that increase binding include large protein size, amino acid sequences that are rich in proline, and a lack of protein structure [28]. Due to their specific preference for tannin binding, PRP’s have been studied in disciplines that have aimed to understand adaption to seasonally tannin-rich diets in animals [29–32]. Salivary PRPs have been of interest in sensory studies due to their ability to irreversibly precipitate tannins, contributing to the sensation of astringency [28]. Tannin-PRP complexes have been found to be insoluble throughout the gastrointestinal tract [1, 30], thus complexes are maintained during digestion. Moreover, like non-heme iron absorption, PRP profiles tend to show high intrapersonal, rather than interpersonal variability [33], that is genetically linked [34, 35], which may explain why some individuals have enhanced capacity for ‘antinutritional’ consumption without impaired iron status. Increases in PRP secretion with tannin consumption have been shown to improve protein [36], and non-heme iron availability [37, 38] in rats, while hamsters without capacity to enhance PRP synthesis tannin consumption resulted in poorer protein availability [31, 38].

This systematic review aims to explore whether tannin-PRP complexes may reduce tannin-non-heme iron chelation during digestion, and to determine whether biochemical mechanisms behind tannin-PRP binding could reduce tannin-non-heme iron chelation during digestion. This review will identify in vitro and in vivo research exploring biochemical mechanisms and outcomes related to tannin-PRP binding and non-heme iron. Secondary aims are to compare potential differences in non-heme iron bioavailability attributed to hydrolyzable versus condensed tannins PRP-binding mechanisms in order to explore whether food, or condensed tannins, may affect non-heme iron chelation differently than hydrolyzable tannins commonly used in non-heme iron absorption studies.

## Methods

### Primary outcomes and search strategy

Primary search subjects included non-heme iron and/or salivary proline-rich proteins exposed to tannins, both condensed and hydrolyzable. Search outcomes were targeted toward research exploring biochemical modeling for binding methods, binding strength, mechanism of binding comparatively, effects of binding of one compound on another, and long-term effects of tannin consumption on PRP and non-heme iron availability. Studies were not excluded for sample or effect size. To capture as many relevant citations as possible, medical and scientific databases were used (Pubmed, Web of Sciences Core Collection, Cochrane Database, Medline, Proquest Nursing, and CABI), as well as snowball article collection (citations from relevant journal articles) and internet search engines (Google Scholar) to look for other references. All publication dates were included and searches were completed by July 2016. Articles reviewed were in English.

### Search terms and inclusion

Article searches in PubMed included (“Salivary Proline-Rich Proteins”[Mesh] AND “Non-heme iron”[Mesh]) AND “Tannins”[Mesh];(“Salivary Proline-Rich Proteins”[Mesh] AND “Non-heme iron”[Mesh]) 2012; (“Salivary Proline-Rich Proteins”[Mesh] AND “Tannins”[Mesh] 2009–2014; (“Non-heme iron”[Mesh]) AND “Tannins”[Mesh] 1945–2015; (“Salivary Proteins and Peptides”[Mesh]) AND “Non-heme iron”[Mesh] 983–2015; ((“Salivary Proteins and Peptides”[Mesh]) AND “Non-heme iron”[Mesh]) AND “Tannins”[Mesh];(“Proline-Rich Protein Domains”[Mesh]) AND “Tannins”[Mesh] 2009–2016;“Proline-Rich Protein Domains”[Mesh] AND “Non-heme iron”[Mesh] (“Tannins”[Mesh]) AND “Salivary Proteins and Peptides”[Mesh] 1983–2015; (salivary protein[All Fields] OR salivary proteinase[All Fields] OR salivary proteins[All Fields]) AND (“non-heme iron”[MeSH Terms] OR “non-heme iron”[All Fields]) 1966–2016; salivary[All Fields] AND (“proline”[MeSH Terms] OR “proline”[All Fields]) AND rich[All Fields] AND (“proteins”[MeSH Terms] OR “proteins”[All Fields] OR “protein”[All Fields]) 1973–2016. Filters included searches for in vivo*,* in vitro*,* human and animal models. Web of Science Core Collection and CABI search terms included “salivary proline rich proteins AND tannins (condensed OR proanthocyanidins AND/OR hydrolyzable) AND/OR non-heme iron”; “tannins AND non-heme iron” (All years). Proquest nursing and MEDLINE (all years; terms included: “salivary proline rich protein*” AND proanthocyanidins; “salivary proline rich protein*” AND non-heme iron; (“salivary protein*” AND non-heme iron) AND tannin; (“salivary protein*” AND tannin); (“salivary protein*” AND non-heme iron) (All years). Cochrane search terms were: #1 MeSH descriptor: [Non-heme iron] explode all trees 1785 #2 MeSH descriptor: [Proanthocyanidins] explode all trees; #3 MeSH descriptor: [Salivary Proline-Rich Proteins] explode all trees (all years).

### Selection, quality assessment, data extraction and analysis

**Fig. 2:**
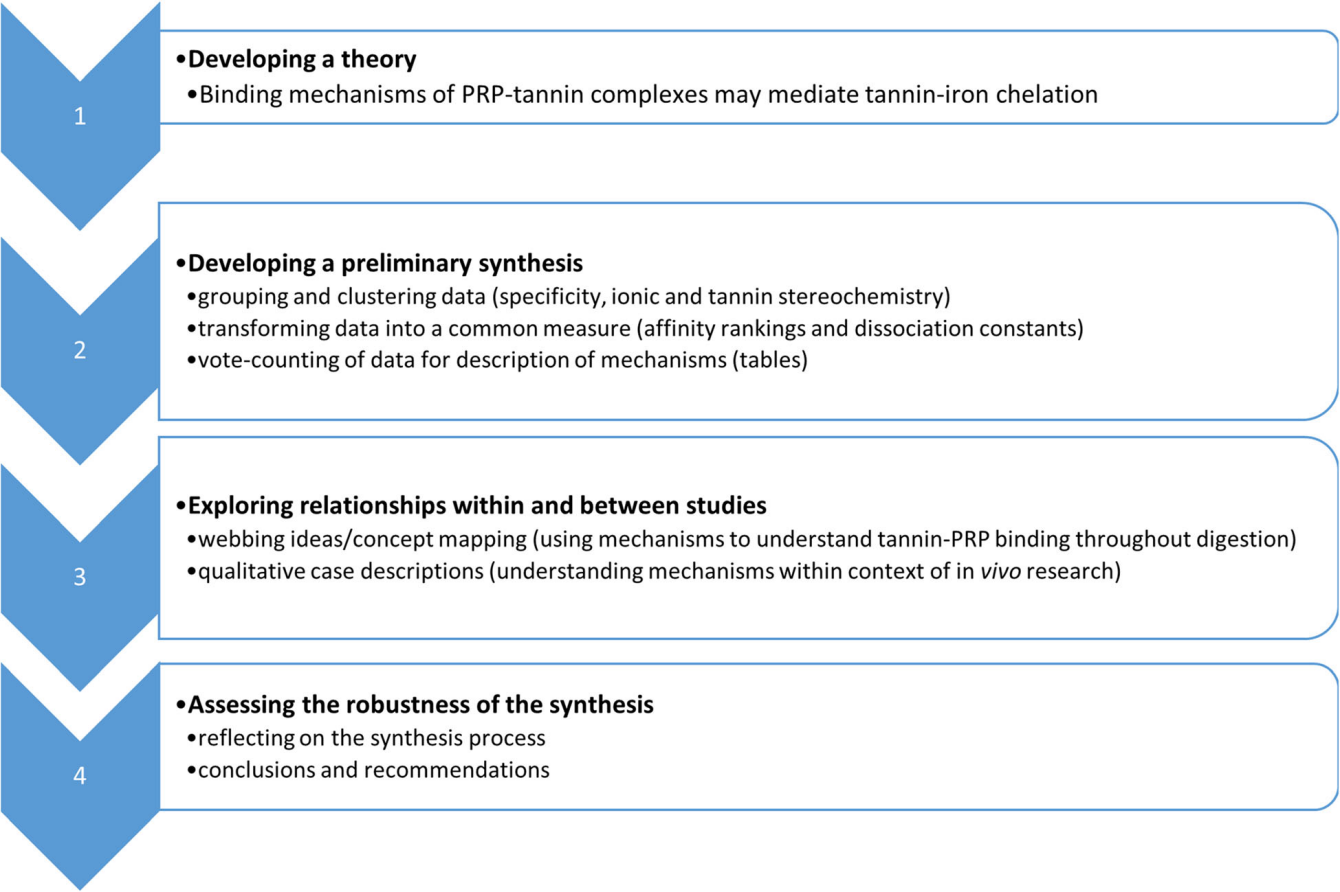
Data analysis process for narrative review of tannin-non-heme iron and tannin-PRP interactions, adapted from the Center for reviews and dissemination [41]

Articles retrieved were collected into an online database, and duplicates were removed. Remaining articles were included with the presence of at least two key terms, and then by abstract reviews for relevance to study outcomes. After initial inclusion criteria were met, articles were collected for full review. Quality assessment of data was completed using the Cochrane Quality Guide [39] adapted for in vitro studies (Additional file 1). In brief, articles were reviewed for risk of bias based on methodology, results presented and discussion. They were classified as having high, unclear, or low risk of bias based on their overall study characteristics. Studies with overall high or unclear risk of bias were excluded from the review. Data from remaining articles were collated into summary tables (biochemical study characteristics, Additional file 1). For biochemical binding analysis, a narrative synthesis approach was chosen due to the heterogeneity of studies explored and lack of studies exploring tannin-non-heme iron: tannin-PRP binding comparisons. This process included developing a theory of how study interventions worked, developing a preliminary synthesis of findings in included studies, exploring relationships within and between studies, and assessing the robustness of the synthesis formulated (Fig. 2) [40]. For analysis, studies were grouped primarily by study question, then by subgroup topics.

For in vivo analysis, study outcomes were collected, mean outcome measurements and standard deviations were used to calculate Hedges’ *g* estimates, and results were used to calculate effect size [41]. Upper and lower confidence limits of effect size were calculated using a 95% confidence interval, and total effect size was calculated from normalized values [41]. PRP synthesis relative to control gave reference to PRP production with non-heme iron-tannin outcomes using the equation:

$$ Relative\  PRP\  expression=\frac{Average\  in crease\kern0.75em  in\  PRP\  production\ (intervention)}{Average\  in crease\kern0.75em  in\  PRP\  production\ (control)} $$

## Results

### Inclusion criteria

**Fig. 3:**
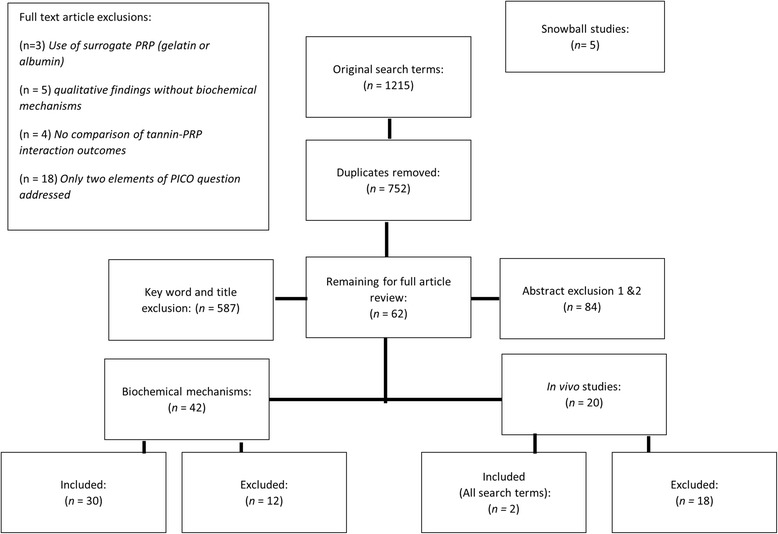
Inclusion and exclusion criteria for review. Articles were excluded that did not include key terms, that were not comparative studies, and for models that were biologically dissimilar to saliva. *PRP* proline-rich proteins

Original search terms generated 1220 articles that were added into an electronic database (Fig. 3). After duplicates were automatically removed, 752 articles were reviewed for title, abstract, and key term relevance. Articles were removed without presence of at least two key terms in abstracts (one of two key subject terms; non-heme iron and/or salivary proline-rich proteins) AND an intervention term (tannin exposure). Excluded studies at this stage included validity studies for measurement tools, assessment studies (including tannin content of foods, food non-heme iron content), studies in ruminant animals, research exploring topic subjects in specialized conditions (burning mouth syndrome, Sjogren’s syndrome, Additional file 1). The 107 articles remaining were manually combed for duplicates, and a second review of abstracts for specific population intervention comparison and outcomes (PICO) specified further narrowed articles. Articles were obtained for full article review from online journal archives, printed journals, and interlibrary loan. Because of the heterogeneity of remaining studies available to answer the research question, full text review of the remaining 81 articles was divided into two groups: biochemical mechanisms for tannin binding, and in vivo comparisons of non-heme iron status with tannin consumption and PRP production. For biochemical mechanisms, articles were excluded that did not address PRP interactions with tannins specifically. This exclusion criterion was applied due to suggestion that PRPs have different binding capacity and mechanisms than other proteins, limiting efficacy of substitute models [42]. Other reasons for exclusion at this stage were that articles did not include biochemical mechanisms explaining tannin-PRP binding. Remaining in vivo articles were excluded that did not nclude all three outcomes related to the study question: non-heme iron, tannins, and salivary PRPs. Full text review of articles was completed including analysis for internal and external bias. Final analysis included 32 studies (for biochemical modeling: 30 in vitro and 2 in vivo animal studies).

### Part I: Mechanisms behind tannin-PRP binding, effects of tannin and PRP characteristics on binding affinity

Of studies reviewed, three themes potentially important to considering binding and affinity of PRPs to tannins were identified. The first was that tannin-PRP binding mechanisms are specific, and that binding affinity and efficiency are affected by tannin and PRP concentration. Next was that ionic and digestive influences favor tannin-PRP complex formation throughout digestion. Finally, the stereo chemical makeup of tannins and PRPs themselves is important to preferential binding and affinity of tannin-PRP connections.

#### Tannin-PRP binding specificity

**Fig. 4:**
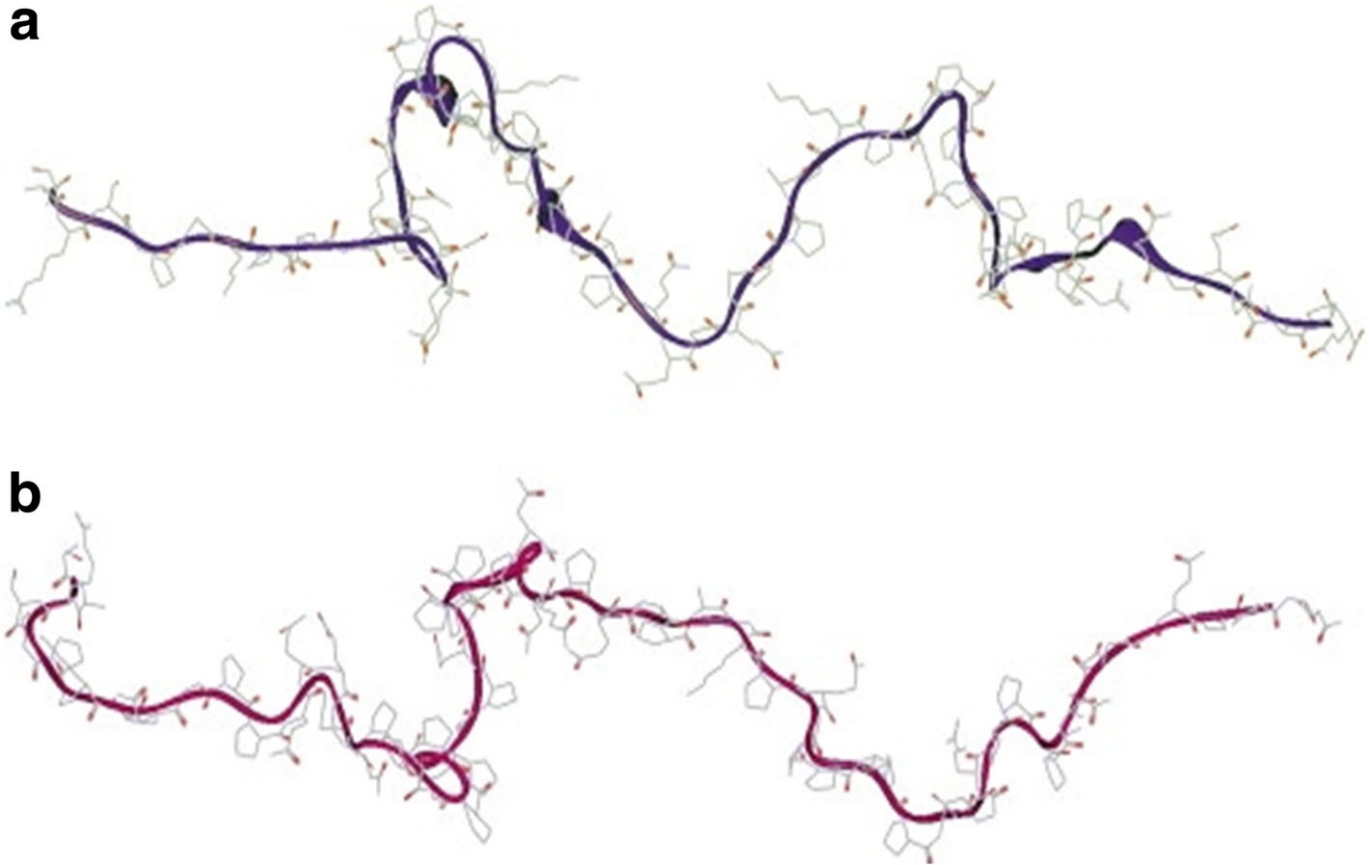
*Open* and *flexible* conformations of a basic PRP molecule with proline residues [47], with permissions

PRPs are randomly structured, unfolded proteins [43] where open and flexible conformation of helical and extended coils allows for binding of tannins to proline-rich residues (Fig. 4) [44–46]. Interactions between PRPs and tannins first occur through hydrogen bonds between compounds enhanced by glycine, arginine, and alanine [43, 45, 47]. It is important to note that PRPs bind specifically to tannins [43, 47–52] (Table 1), and with an increased affinity compared to digestive enzymes and proteins [4, 53], which supports the role of PRPs serving as biological protectors against tannins. Selectivity is regulated by both tannin and PRP properties enhancing binding affinity; high-affinity tannin molecules are bound first, then lower affinity molecules are bound at higher concentrations [54]. Enzyme activity inhibited by tannins is enhanced in the presence of PRPs, pointing to tannin-PRP preferential binding [52], and similarly structured compounds to PRPs have not been found to bind to tannins with the robust affinity found in tannin-PRP binding [50].

**Table 1:** Tannin-PRP binding specificity

Reference	Method	Tannin type	PAC-TA comparison?	Conditions of assay	Outcome	Mechanism agreement
[48]	NMR	B2, PGG, TGG, PAC monomer, epicatechin	yes	40 mM B2, other assays 50 mM; either 0.5 ml of 2 mM or 4 mM PRP from mouse; pH 3.8	N-terminal proline residues linked to amide and amino structures bind tannin, then secondary interactions with galloyl groups changes structure of open conformation around PRP (specific binding)	Yes
[49]	NMR	PAC as B1, B3, C2	No	0.5-20 mM PRP (IB9) with 15.7 mM tannin; pH 3.5	Tannin-PRP binding is specific to a certain concentration of tannin; then becomes random	Yes
[50]	ESI-MS	EgCG, ECG, B2, B2 3-O gallate, reserpine	No	1:10 ratio protein: polyphenol; pH 3.2	Tannin-PRP binding is specific; PRP-reserpine did not bind (similar structure to studied tannins)	Yes
[51]	ESI-MS; DLS, SAXS	EgCG	No	0.336 mM (1–3.5 mg/ml) PRP (IB5); 2:1 protein: polyphenol; pH 5.5	Tannin-PRP interaction is specific and dependent on tannin interactions; PRP sites for tannin binding are independent and have free energy; at a threshold, multidendate tannin crosslinks strengthen tannin-PRP bonds	Yes
[43]	DLS, ITC	EgCG	No	6.4 or 12.8 EgCG with 0.25–2 mg IB5; pH 3.5	Tannin-PRP interaction is concentration dependent; there is slow and specific binding of tannins followed by rapid and non-specific aggregation as tannin-PRP binding sites are saturated.	Yes
[52]	In vitro digestion, SDS-PAGE, HPLC	EgCG	No	0.05–0.5 mM EgCG, Human salivary PRPs; protein: tannin ratio 3:1; pH gastric 2.07; duodenal pH 7.8	Preferential tannin-PRP binding compared to lipase, alpha amylase, chymotrypsin, trypsin, lactase	Yes
[86]	DLS, ITC	EgCG	No	1:5 ratio saliva: wine in 1% TFA compared to physiological conditions	Salivary PRP ‘moderately’ bound tannins	Yes
[47]	NMR, DLS	EgCG, EGC, PGG	Yes	20 mM polyphenol with 2 mM mouse PRP; pH 3.8	There is preferential binding of tannin to proline residues of PRPs vs. alternative amino acids	Yes

*NMR* Nuclear magnetic resonance imaging, *ESI-MS* electrospray ionization mass spectrometry, *DLS* dynamic light scattering, *SAXS* small angle X-ray scattering, *ITC* isothermal titration calorimetry, *SDS-PAGE* sodium dodecyl polyacrylamide gel electrophoresis, *HPLC* high performance liquid chromatography. B1, B2, B3: proanthocyanidin B1, B2, B3, *PGG* pentagalloylglucose, *TGG* tetragalloylglucose, *PAC* proanthocyanidin, *EgCG* epigallocatechin gallate, *ECG* epigallocatechin, *PRP* proline-rich protein, *TA* tannic acid

As tannins bind to and saturate proline-rich residues, their multidendate, or self-associating nature [51], leads to cross-linked binding between tannin molecules, formation of hydrophobic tannin-PRP bonds [43], and more efficient and stable precipitation of tannin-PRP complexes [49, 51]. The concept of affinity and eventual tannin-PRP precipitation may explain the effectiveness of tannin-PRP complexes in preventing tannin-iron chelation. In a non-precipitated state, tannins are highly bound to PRPs, however, lack of saturation of proline-rich residues increases the likelihood that tannin-PRP complexes will dissociate [43, 51]. This may lead to tannin-iron chelation later during digestion. Tannin-PRP binding at low tannin concentrations may follow a ‘poisoned growth model,’ wherein aggregation of tannins through crosslinking is limited by PRPs that do not have sufficient saturated proline residues to continue the process [51]. In this model, a lack of saturated proline-rich residues on PRPs favors redistribution of tannin molecules among all PRPs, reducing the crosslinks formed by tannins and leading to dissociation and ‘freeing’ of tannins from PRPs [51].

#### Concentration effects

High tannin concentrations support almost immediate formation of hydrophobic bonds and tannin crosslinks with PRPs, along with subsequent precipitation [43]. In contrast, lower tannin concentrations resist precipitation due to lack of tannin crosslinking and hydrophobic bonds in favor of weaker hydrogen bonds that may later dissociate (Table 2) [47, 51, 53, 55–59]. Thus, as concentration of tannin increases, PRPs have the ability to bind tannin molecules beyond the number of proline residues that they contain [47, 51], leading to more efficient tannin ‘capture’ by PRPs. After concentration-dependent hydrophobic interactions and tannin-crosslinking is established, conformational changes in the PRP molecule provides additional stability of tannin-PRP complexes, further reducing likelihood of digestive dissociation [48, 60].

**Table 2:** Effects of tannin, PRP concentration on binding

Reference	Method	Tannin type	PAC-TA comparison	Conditions of assay	Outcome	Concentration effect
[53]	DLS	PAC as tetramers, pentamers, gallates	No	31.2 mg/L GSE: 0.5–5 mg/L IB-8c or 3.12 mg/L IB-8c: 19.5–46.8 mg/L GSE; pH 5.0, 12% ethanol	Increase in PRP concentration increases aggregation and precipitation of tannins to a maxima, then increased protein concentrations favors dissociation due to reductions in tannin-cross linking	Tannin stacking and crosslinking at higher concentrations
[55]	SDS-PAGE, HPLC, tryptic digestion	PAC as dimers, trimers, tetramers	No	0.00–1.5 mM GSE in saliva; pH 5.0, 12% ETOH	At higher tannin concentrations, less PRP are required for similar binding at lower tannin concentrations.	Tannin stacking and crosslinking at higher concentrations
[56]	ESI-MS	EgCG, ECG, B2, B2 3-O gallate, reserpine	No	1:10 ratio protein: polyphenol; pH 3.2	Higher tannin concentration of tannins favor tannin-PRP stability in gastric digestion	Stability of tannin-PRP binding
[51]	ESI-MS; DLS,SAXS	EgCG	No	0.336 mM (1–3.5 mg/ml) PRP (IB5); 2:1 protein: polyphenol; pH 5.5	At lower concentrations, PRP are bound to tannins, but soluble. At higher concentrations, more tannin is needed to effectively bind the same amount of PRP; this happens as binding occurs regardless of proline terminal residue numbers.	Tannin stacking and crosslinking at higher concentrations
[57]	HPLC-DAD	PAC as monomers, dimers, trimers	No	1–8 ml saliva mixed with 40 ml GSE or 20 or 40 ml sipped	Increased tannin concentration increases precipitation.	Tannin stacking and crosslinking at higher concentrations
[58]	ITC	GSE as catechin, epicatechin, epicatechin 3-O gallate	No	5–25 μg PAC and 40 μl saliva with 10% ethanol	Increased tannin concentration increases precipitation.	Tannin stacking and crosslinking at higher concentrations
[47]	NMR, DLS	EgCG, EGC, PGG	Yes	20 mM polyphenol with 2 mM mouse PRP; pH 3.8	The number of PRP binding sites does not correlate with the corresponding decrease in tannin concentration after tannin-PRP binding at higher concentrations	Tannin stacking and crosslinking at higher concentrations
[59]	NMR	Tannic acid	No	1:0–1:5.6 ratio of PRP to tannic acid	More tannin-PRP complexes that are bound, the less that the complexes dissociate	Stability of tannin-PRP binding

*NMR* Nuclear magnetic resonance imaging, *ESI-MS* electrospray ionization mass spectrometry, *DLS* dynamic light scattering, *SAXS* small angle X-ray scattering, *ITC* isothermal titration calorimetry, *SDS-PAGE* sodium dodecyl polyacrylamide gel electrophoresis, *HPLC* high performance liquid chromatography. B1, B2, B3: proanthocyanidin B1, B2, B3, *PGG* pentagalloylglucose, *TGG* tetragalloylglucose, *PAC* proanthocyanidin, *EgCG* epigallocatechin gallate, *ECG* epigallocatechin, *PRP* proline rich protein, *TA* tannic acid

**Fig. 5:**
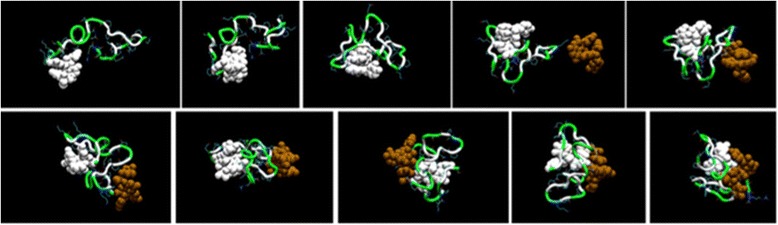
Canon et al. [50], with permission: condensed tannin (*white* and *brown*) interaction with PRP peptide (*green ribbon*) during molecular dynamics run. Tannins associate with PRP molecules and attach to proline rich residues through hydrophobic bonds. On binding to PRPs, the multidendate nature of tannins allows for hydrophobic bond formation and conformational changes in the PRP molecule to stabilize the complex

Thus, the process of tannin-PRP binding is proposed in three stages: first, proline residues are saturated by tannins that bind selectively, and hydrogen bonds strengthen the tannin-PRP complex. Next, tannins self-associate and hydrophobic stacking of multiple tannins promotes crosslinking of tannin-PRP complexes via tannin-tannin bonds, and conformational PRP changes stabilize the bond. Last, tannin-PRP complexes aggregate and separate from solution (precipitation, Fig. 5) [43, 49].

#### Ionic effects

Ionic concentration and solvent effects also affect tannin-PRP binding affinity. Hydrophilic [53] and basic [52, 59, 61–63] environments tend to reduce tannin-PRP affinity, partly due to reductions in hydrogen and hydrophobic binding [53, 63] (Table 3). Potential of hydrogen (pH) in gastric compared to enteric environments significantly increases precipitation of PRPs [62], and lower ionic concentrations may inhibit secondary structural changes needed for PRP-tannin association [51, 64]. Tannic acid is more soluble in acidic environments [59], suggesting that precipitation may be necessary for PRP-induced protective mechanisms against tannins. Food matrix components, carbohydrates and ethanol particularly [53, 63], disrupt precipitation of tannin-PRP aggregation, necessitating increased concentrations of tannins to precipitate PRPs. It is important to note that matrix disrupted interactions may limit tannin-iron exposure as well.

**Table 3:** Effects of pH on tannin-PRP solubility

Reference	Method	Tannin type	pH	Effect
[61]	Diffusion precipitation interaction assay	Hydrolyzable and condensed wine extracts, catechin, tartaric acid, gallic acid	3.5 vs. 7.0	↓pH ↑precipitation
[62]	Competitive binding assay	Quebracho and tannic acid	2.0 vs. 7.4	↓pH ↑precipitation
[52]	In vitro digestion precipitation interaction assay, HPLC, SDS-PAGE	EgCG	2.07, 7.8, 5.0–9.0	↓pH + ↑ tannin = enzyme inhibition, blunted by PRP
[59]	NMR	Galloyl rings from tannic acid	3.5 vs. 1.7	↓pH ↑precipitation
[63]	SDS PAGE	Wine or tannic acid	2.9, 3.0, 3.6	↓pH ↓solubility of tannin-PRP

*NMR* Nuclear magnetic resonance imaging, *SDS-PAGE* sodium dodecyl polyacrylamide gel electrophoresis, *HPLC* high performance liquid chromatography, *EgCG* epigallocatechin gallate, *PRP* proline rich protein

#### Digestive effects

In vitro digestion experiments have shown that tannin-PRP bonds are highly resistant to digestion [52, 55, 56, 62, 65]. Tannin-PRP bonds are resistant to trypsin cleavage [55], and in the presence of enteric digestive protease enzymes, tannin-PRP complexes are more likely to stay insoluble than in their absence [62]. Gastric digestion and pH favor precipitation of tannin-PRP aggregates [52, 55, 56, 62, 65], although smaller tannin size and lower concentration reduce stability in gastric digestion [56]. PRPs are also highly bound and recovered in enteric digestion [56], and may block enteric absorption and digestion of tannins themselves [52, 62]. Specific binding affinity of tannin-PRP complexes seems to occur outside of the mouth; addition of PRP to hydrolyzable tannin has been found to reduce tannin absorption 2–3 fold in Caco-2 cells [65].

#### PRP characteristics and tannin interactions

Many PRP classifications have been identified in saliva, and are grouped according to their biochemical properties. When referencing their ability to precipitate tannins in whole saliva, acidic PRPs (aPRP) tend to bind to tannins with the highest affinity, followed by basic (bPRP), and glycosylated PRPs (gPRPs) [55, 56]. While tannin-PRP affinity may explain the predilection for complex formation, this does not always correlate to the ability to *efficiently* precipitate tannins. Greater proline content in bPRP [62, 66], and gPRP [66] is associated with increased ability to precipitate higher concentrations of tannins compared to aPRPs. Longer PRP sequences found in bPRP and gPRP with more proline-rich residues result in more tannin-proline rich residue interactions [67], and allow for secondary conformational changes around tannin molecules [47], likely reducing complex dissociation compared with shorter PRP sequences [45]. Glycosylation may increase the threshold for precipitation of tannins without impeding tannin-PRP affinity [58], although this finding has been inconsistent [68].

In vivo*,* PRP profiles differ among regular tannin consumers and non-consumers. For example, gPRP have been found more prevalently in tannin consumers’ salivary profiles, and tannins are precipitated at a lower rate (30%) in consumers than in non-consumers (85%), possibly due to increased capacity of PRP types produced [4]. Types of PRPs that were precipitated also varied between tannin and non-tannin consumers. Tannin consumer PRP profiles consisted of higher prevalence of bPRP and gPRP, which were most commonly precipitated [4].

#### Tannin polymerization, galloylation, and hydroxylation

Tannin polymerization, galloylation, and hydroxylation play a role in tannin-PRP affinity and precipitation (Table 4). Increased polymerization, or increases in the relative size of tannin molecules, may favor tannin-PRP affinity through hydrophobic interactions and subsequent increases in self-association of tannin molecules to form precipitating complexes [48]. Increased galloylation [66, 69], as well as hydroxyl group positioning [50, 67], may increase affinity, as well as binding strength of tannins to PRPs through promotion of hydrogen bonds. Perhaps less importantly, the location of carbon-carbon bonds within polymers may improve stability of precipitates formed by tannin-PRP complexes [69].

**Table 4:** Effect of tannin polymerization, galloylation, and hydroxylation on PRP affinity and precipitation

Reference	Method	Tannin type	Polymerization (1)	Galloylation (2)	Hydroxylation of the B ring (3)
[50]	ESI-MS	EgCG, ECG, B2, B2 3-O gallate	↑↑↑	↑↑	↑
[48]	NMR	B2, PGG, TGG, PA monomer	↑↑↑	↑↑	*nd*
[69]	nephelometry	B1–9, C1, B2 3-O gallate, E	↑↑↑	↑↑	*nd*
[49]	NMR	PAC as B1, B3, C2	↑↑↑	↑↑	↑
[67]	ESI-MS	B1, B2, B3, B4, C2, C, E, quercetin derivatives	↑↑↑	*nd*	↑↑
[56]	ESI-MS	EgCG, ECG, B2, B2 3-O gallate, reserpine	↑↑↑	*nd*	*nd*
[58]	ITC	GSE as catechin, epicatechin, epicatechin 3-O gallate	↑↑↑	*nd*	*nd*
[66]	Competitive binding assay	5GG, gallic acid, EGC	*nd*	↑↑↑	*nd*

*NMR* Nuclear magnetic resonance imaging, *ESI-MS* electrospray ionization mass spectrometry, *ITC* isothermal titration calorimetry; B1, B2, B3, B4, C1: proanthocyanidin B1, B2, B3, B4, C1, *PGG* pentagalloylglucose, *TGG* tetragalloylglucose, *PAC* proanthocyanidin, *EgCG* epigallocatechin gallate, *ECG* epigallocatechin, *PRP* proline rich protein, *nd* not determined

Tannin polymerization affects tannin-PRP affinity and dissociation more than either tannin galloylation or hydroxylation [49, 50]; larger tannin molecules precipitate PRPs more efficiently [48–50, 56, 59, 67, 69], and selectively [4], than smaller molecules. Smaller molecules do bind PRPs, but do not crosslink [69], and the lack of tannin self-association increases the likelihood that tannin-PRP complexes dissociate [47, 51, 56, 62, 66]. In vivo*,* tannin polymerization has been shown to be positively correlated with precipitation; however, tannin consumers’ saliva is able to precipitate smaller tannins, and given that greater polymer size is bound preferentially, does not leave tannins unbound. Non-consumers, on the other hand, may bind only large tannins [4].

#### Binding of hydrolyzable vs. condensed tannins

PRP binding to hydrolyzable versus condensed tannins is influenced both by differences in binding affinity [47, 61, 62, 66] and subsequent likelihood of bond dissociation during digestion [47, 48, 62, 63] (Table 5). PRP affinity toward hydrolysable tannins is greater than condensed tannins [47, 61, 62, 66]. Affinity of hydrolyzable tannins toward PRPs may reduce the number of available proline residues for tannin-binding compared with condensed tannins [47, 62, 66]. It has been noted that there is a greater stability of PRP with quebracho (a condensed tannin), measured by precipitation, in both gastric and enteric conditions compared to tannic acid [62]. This may be due to weaker hydrogen bonds that hold hydrolyzable tannin-PRP complexes together [47, 48] compared with more prominent hydrophobic bonds found in condensed tannin-PRP precipitates [63]. The ring structure in condensed tannins may favor a more stable hydrophobic bond compared with hydrolyzable tannins [69]. It has been suggested that hydrolyzable tannins may not crosslink compared to their condensed counterparts [48], reducing bond stability and increasing likelihood of complex dissociation. Differences in binding mechanisms described above may also reduce the physiological stability of hydrolyzable compared to condensed tannin-PRP complexes throughout the digestive process [47, 48, 62, 63].

**Table 5:** PRP binding to hydrolyzable vs. condensed tannins

Reference	Method	Tannin type	Bond stability	Binding affinity
[66]	Competitive binding assay	5GG, gallic acid, EGC	*nd*	Hydrolyzable tannin > condensed tannin
[62]	Competitive binding assay	Quebracho and tannic acid	Hydrolyzable: 20% greater dissolution of bonds in gastric and enteric digestion	Hydrolyzable tannin > condensed tannin
[48]	NMR	B2, PGG, TGG, PAC monomer	Hydrogen bonds associated with hydrolyzable tannins	B2 > PGG > TGG>
[47]	NMR, DLS	EgCG, EGC, PGG	Hydrogen bonds associated with hydrolyzable tannins	Hydrolyzable tannin > condensed tannin
[63]	SDS PAGE	Wine or tannic acid	Condensed tannins associated with hydrophobic bonds	*nd*
[61]	HPLC	Hydrolyzable vs. condensed wine extracts	*nd*	Increased precipitation of condensed tannin at pH 7.5 c/t hydrolyzable tannin

*NMR* Nuclear magnetic resonance imaging, *DLS* dynamic light scattering, *SDS-PAGE* sodium dodecyl polyacrylamide gel electrophoresis, *HPLC* high performance liquid chromatography. B2: proanthocyanidin B2, *PGG* pentagalloylglucose, *TGG* tetragalloylglucose, *PAC* proanthocyanidin, *EgCG* epigallocatechin gallate, *ECG* epigallocatechin, *PRP* proline rich protein. *nd* = not determined

### Part II: Effect of tannin consumption on PRP expression and non-heme iron bioavailability

#### In vivo comparisons

Two studies have explored the interaction of chronic tannin ingestion, PRPs and non-heme iron bioavailability [37, 70] (Table 6). Due to the similarities of the two studies, it is possible to compare effects of several treatments to control conditions in order to isolate findings for synthesis. Thus, PRP production and the effects of tannin consumption on non-heme iron absorption versus hepatic iron status are highlighted. Further, isolation of experiments employing chronic ingestion of tannins is included to give context to effect of tannin ingestion over time.

**Table 6:** In vivo studies comparing PRP expression and non-heme iron availability with tannin ingestion

Study	Model	Intervention	Study conditions	Tannin concentration in challenge diet (g/kg)	Fe(diet)	n	Study duration	Measurement of bioavailability	PRP measurement
[70]	Sprague Dawley rats	Green tea	Control (acute and chronic)	28.6 g/kg green tea (nd)	20 mg/kg	6	7, 30 days	% absorption (^59^Fe test meals), Hepatic non-heme iron	SDS-PAGE, MALDI-MS/MS,DIGE
			Acute gastric gavage			6	7 days		
			Chronic gastric gavage			6	30 days		
			Acute tea powder			6	7 days		
			Chronic tea powder			6	30 days		
[37]	Sprague Dawley rats	Black tea	Control	0.3 ± 0.0	35.9 ± 1.3	6	24 days	% absorption (^59^Fe test meals), Hepatic non-heme iron	SDS-PAGE
			5% tea solids challenge tannin free diet	8.9 ± 0.5	35.9 ± 1.3	6	24 days		
			5% tea solids +6% gelatin challenge tannin free diet	9.9 ± 0.7	35.9 ± 1.3	6	24 days		
			5% tea solids chronic diet	8.9 ± 0.5^a^	34.3 ± 1.5	6	24 days		
			5% tea solids +6% gelatin chronic	9.9 ± 0.7^a^	34.6 ± 1.0	6	24 days		

^a^Supplied as 25 g/kg in diet

*SDS-PAGE* sodium dodecyl polyacrylamide gel electrophoresis, *MALDI-MS/MS* Matrix-assisted laser desorption/ionization/mass spectrometry, *DIGE* difference gel electrophoresis

### Study characteristics

Both of the studies reviewed included a diet challenge after either acute or chronic ingestion of a tannin-rich diet in weanling Sprague-Dawley rats, however they employed different diet mechanisms to attempt to explore the effect of PRP expression on non-heme iron absorption and hepatic iron stores. The first (Study 1) study provided tannin-containing green tea powder or a control diet, fed for 7 or 30 days, compared with a tannin-containing gavage (green tea) diet. In the gavage diet, exposure of tannin to the oral cavity was bypassed with the goal to reduce expression of PRPs [70]. This study included a diet challenge at either day 1 (acute) or day 24 (chronic) to measure non-heme iron absorption, after collection of baseline bloodwork. Green tea powder and control diets were given as powdered diets, with or without green tea powder; animals in these groups were given a twice daily phosphate buffered saline gavage. Green tea gavage groups were given a control powdered diet with green tea gavages twice daily throughout the study [70]. Tannin concentration in gavage diets was based on oral tannin consumption of green tea diets by rats.

The second (Study 2) study included a control or tannin-containing diet (as black tea, half the amount of tannin given at challenge) with tannin challenge at midpoint (day 11) for all groups. Substitution of protein with gelatin (proline-rich) in separate chronic and acute ingestion groups aimed to assess the effects of PRPs on non-heme iron absorption with proline addition [37]. In study 2, intervention groups were given a diet with brewed black tea that was made into a slurry with powdered diets. Slurry diet components were then freeze dried and milled into powdered diets [37]. Diets consumed in both studies contained similar tannin and iron content (Table 6). Study 2 diet intake was not significantly different throughout the study, but was about 3 times less in the chronic intervention (tea) group on day 1-3 compared to the rest of the study. Both studies used a 2 g test meal for iron absorption, which was totally consumed by rats.

### Comparison I: Effect of tannin on non-heme iron absorption and stores with acute and chronic tannin ingestion

Data are presented in means ± SEM. Study 1 hepatic iron stores were not significantly different among treatment groups (*p* = 0.521) [70]. Acute treatment outcomes were not different from chronic outcomes in control (53.5 ± 11.1 vs. 61.0 ± 4.0 μg Fe/g liver), gavage (58.4 ± 2.0 vs. 57.6 ± 7.6 μg Fe/g liver), or green tea powder (47.2 ± 2.7 vs. 54.2 ± 7.5 μg Fe/g liver) groups [37] (Table 7). Study 2 hepatic iron stores were not significantly different among treatment groups (*p*-value not presented). Acute and chronic groups’ hepatic iron levels were not significantly different (58.5 ± 5.2 and 58.8 ± 6.9 vs. 43.5 ± 3.4 and 46.6 ± 3.6 μg Fe/g liver) in challenge and long-term tea and gelatin diets, respectively [37]. Calculated confidence intervals supported that overall, there was a non-significant, but positive, effect of tannin consumption on hepatic iron stores (calculated effect size *d =* 0.57, 95% CI -0.64 to 1.84 in favor of treatment; Table 7). This trend was more positive in chronic tannin consumers (calculated effect size *d =* 1.16, 95% CI -0.18 to 2.51 in favor of treatment).

**Table 7:** Effect size of acute and chronic hepatic iron levels

Study	Treatment	Effect size (*d)*	Lower CI (95%)	Upper CI (95%)
[70]	Acute gavage	0.17	−0.96	1.31
	Chronic Gavage	−0.20	−1.33	0.94
	Acute Powder	−0.22	−1.36	0.91
	Chronic Powder	−0.40	−1.55	0.74
[47]	5% tea challenge	−0.34	−1.48	0.80
	5% tea chronic	2.87^a^	1.26	4.48
	5% tea + gelatin challenge	0.50	−0.64	1.65
	5% tea + gelatin chronic	2.39^a^	0.91	3.87
Total (CI % hepatic iron)		0.57	−0.64	1.84
Total (CI % chronic hepatic iron)		1.16	−0.18	2.51
Total (CI % acute hepatic iron)		0.03	−1.11	1.17

^a^ *p* < 0.05 (95% CI)

**Fig. 6:**
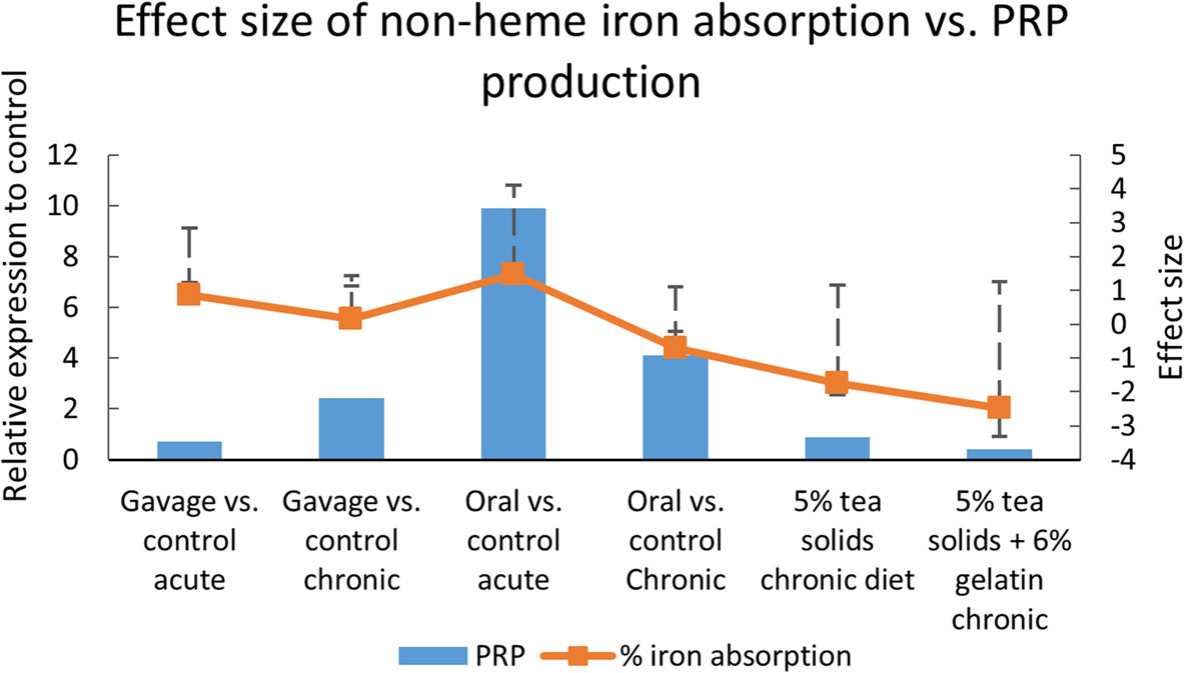
Relative PRP induction compared to non-heme iron absorption effect size. PRP expression follows iron absorption with the exception of oral bypass of tannins in gastric gavage groups

Data for non-heme iron absorption are also presented in means ± SEM. Non-heme iron absorption percentage was not significantly different among treatment groups in study 1 (*p* = 0.104, *p* = 0.292 acute and chronic, respectively). Acute non-heme iron absorption was much higher than chronic non-heme iron absorption in all groups, possibly due to sufficient iron stores in the rats at study end, but absorption among treatment groups were not significantly different in control (43.1% ± 4.9 vs. 5.6% ± 1.0), gavage (56.5% ± 7.5 vs. 6.0% ± 1.0), or oral (63.8% ± 6.4 vs. 3.7% ± 1.2) groups (Table 8, Fig. 6) [70]. Non-heme iron absorption was significantly reduced in Study 2 tannin-consuming groups, although there was significantly greater absorption of non-heme iron from groups consuming tannins over time (7.5% ± 2.3 and 25.1% ± 7.4 vs. 6.8% ± 1.6 and 20.8% ± 4.5) in challenge and long-term tea diet and gelatin diets, respectively, (Fig. 6) [37]. Calculated confidence intervals supported that overall, there was a non-significant but negative effect of tannin consumption on non-heme iron absorption (calculated effect size *d =* −1.30, 95% CI -2.73 to 0.13 in favor of treatment; Table 8). This trend was slightly more positive with chronic tannin consumption (calculated effect size *d = −*1.11, 95% CI -2.37 to 0.16 in favor of treatment).

**Table 8:** Effect size of acute and chronic % non-heme iron absorption

Study	Treatment	Effect size (*d)*	Lower CI (95%)	Upper CI (95%)
[70]	Acute gavage	0.79	−0.38	1.97
	Chronic Gavage	0.14	−0.99	1.27
	Acute Oral	1.37^a^	0.11	2.62
	Chronic Oral	−0.66	−1.81	0.51
[37]	5% tea challenge	−3.99^a^	−5.94	−2.03
	5% tea chronic	−1.62^a^	−2.92	−0.31
	5% tea + gelatin challenge	−4.11^a^	−6.1	−2.11
	5% tea + gelatin chronic	−2.29^a^	−3.74	−0.83
Total (CI % absorption)		−1.30	−2.73	0.13
Total (CI % absorption chronic consumers)		−1.11	−2.37	0.16
Total (CI % absorption acute consumers)		−1.61	−3.08	0.11

^a^ *p* < 0.05 (95% CI)

### Comparison II: Effects of PRP production on non-heme iron bioavailability

While study 1 presented a more detailed analysis of PRP production relative to control, study 2 presented total PRP production for each chronically consuming tannin group and control. Because total PRP production was assessed differently by studies, and is presented in relative amounts compared to control analysis (Table 9). In study 1, total PRP production in oral-tannin ingestion groups was greater than either control or gavage groups [70]. Chronic oral tannin ingestion resulted in reduction of PRP production relative to acute ingestion. In study 2, there were no significant differences in PRP production between control and intervention groups [37].

**Table 9:** PRP production in groups relative to control

Study	Treatment	PRP production/control
[70]	Acute gavage	0.73
	Chronic Gavage	2.4
	Acute Oral	9.9
	Chronic Oral	4.1
[37]	5% tea chronic	1.22
	5% tea + gelatin challenge	1.10

*PRP* salivary proline rich protein

Given that direct tannin precipitation of PRPs would most likely affect non-heme iron absorption with proposed biochemical binding mechanisms, relative PRP production to control was calculated and compared to experimental non-heme iron absorption. Given the somewhat qualitative nature of the findings in both studies, statistical analysis has not been applied. Overall, relative PRP expression is greater in groups that found greater non-heme iron absorption, although this is not true in gavage groups, where PRP induction was low and non-heme iron absorption was generally improved compared with control (Table 8, Fig. 6). Groups where PRP induction was low (chronic tea and chronic gelatin ingestion) trended toward lower non-heme iron absorption.

## Discussion: Comparison of tannin non-heme iron and tannin PRP binding

### PRP tannin binding and affinity

Comparing the nature of tannin-PRP binding elucidates mechanisms that may deter tannin-non-heme iron chelation throughout digestion. Tannin exposure to PRPs in the mouth allows for selective complex formation in lieu of tannin-iron chelation on first contact with the food bolus, and conformational changes, along with tannin-crosslinking, effectively allows PRPs to efficiently bind tannins. The strength of hydrophobic bonds formed with tannin-crosslinking, along with secondary PRP conformational changes with residue saturation, may prevent tannin dissociation during digestion and limit the number of unbound hydroxyl groups with the potential to chelate iron throughout the alimentary tract [52, 60].

Similarly to tannin-PRP binding, non-heme iron species are bound to hydroxyl groups of polyphenols [71], and tannins with greater affinity toward non-heme iron (such as tannins with galloyl groups, or larger tannin-polymers) [72] may bind to tannins more efficiently (and thus fewer tannins chelate more non-heme iron molecules). Because PRPs favor binding to higher order (higher polymerization) polyphenol complexes, it may be that PRPs specifically precipitate tannins most likely to bind to iron during digestion. Beyond the nature of tannin-PRP binding, production of PRPs that better bind tannins (such as bPRP or gPRP) [55, 56] with common tannin consumption [4] may facilitate adaptation to tannin-iron chelation over time.

#### Digestion and ionization

At physiological conditions, tannins tend to be deprotonated [73], forming Lewis bases that are stabilized with Lewis acids, such as Fe^3+^, and to a lesser extent, Fe^2+^ [71, 74]. In acidic conditions, protonated tannins, and can be stabilized after bound ferric iron is reduced to its ferrous state, and reduce another ferric iron molecule [71]. Acidic conditions where iron is easily oxidized therefore favor tannin-iron affinity. Interestingly, gallate complexes oxidize at a much higher rate than catecholate complexes [75], which may be why tannic acid strongly chelates non-heme iron compared with condensed tannin-containing foods. Reductions in iron bioavailability are found at higher pH environments, where polyphenol-non-heme iron complexes prevent Fe^3+^ reduction to Fe^2+^ [76], possibly reducing the amount of non-heme iron that can be taken up by the enterocyte.

Similar tannin-iron and tannin-PRP properties regarding pH may explain why pH strongly influences the nature and strength of tannin-PRP bonds. Condensed tannins and bPRP tend to precipitate within similar, more basic ionic conditions [77]; aPRP tend to precipitate closer to the pH of saliva [55], suggesting that PRPs may have different functions at different stages of digestion [78]. Tendencies of aPRP to precipitate at salivary pH may also favor immediate tannin-PRP binding before non-heme iron can be chelated in the mouth. Importantly, the affinity of tannin-PRP binding is resistant to digestion, thereby reducing exposure of tannins to non-heme iron throughout the digestive tract [52, 55, 56, 62, 65]. Compared with the more basic isoelectric point of condensed polyphenols, tannic acid is deprotonated at a pH closer to 5 [79], where tannin-non-heme iron precipitation is highest [72]. This may explain why tannic acid has more potent affinity and efficient binding than condensed tannins for both PRP and non-heme iron complexes.

#### Hydrolyzable versus condensed tannins

Effective PRP protection against hydrolyzable and condensed tannins may be different due to multiple factors. Tannic acid binds more efficiently to non-heme iron as compared to catechins or food polyphenols [72, 80, 81], increasing efficiency of tannin acid-iron chelation comparatively. As mentioned previously, larger tannin polymers increase non-heme iron-tannin binding [72]. Condensed tannin polyphenols tend to be higher order polymers [56] than tannic acid [65], are more likely to cross-link [48], and less likely to dissociate in digestion. By comparison, tannic acid is not as efficiently bound by PRPs during digestion, increasing the antinutritional potency of these tannins. The overall smaller molecular size of hydrolyzable tannins (especially in tannic acid) decreases non-heme iron binding relative to higher order condensed tannin polymers, but an increased number of galloyl groups on tannins increase affinity compared to condensed tannins [72]. Subsequently, a higher concentration of small monomeric compounds may allow hydrolyzable tannins to more efficiently bind PRPs and non-heme iron, leading to an increased burden on salivary glands to produce PRPs, as well as more effective chelation of non-heme iron when PRPs cannot meet tannin demand. In digestion, the hydrolyzable tannins may more likely dissociate from PRPs [62], further increasing potential non-heme iron chelation compared with their condensed counterparts. In vivo*,* PRP production is more highly upregulated with exposure to a mix of condensed and hydrolyzable tannins compared to tannic acid alone [82], suggesting that PRPs may not be an effective defense mechanism against this compound. It may be important to consider these differences when comparing accommodation of non-heme iron status and availability between food tannins (condensed tannins) and hydrolyzable tannins (tannic acid).

#### Limitations in protective tannin-PRP binding

Despite attributes that potentially favor tannin-PRP binding as a mechanism to prevent tannin-non-heme iron chelation, inefficiencies seem to exist. Similar to tannin-PRP interactions, low concentrations of polyphenols associate with non-heme iron more efficiently than higher concentrations [80]. This phenomenon may importantly explain the inefficiency of tannin-PRP complexes in preventing tannin-non-heme iron chelation, especially in adaption. Following the ‘poisoned growth model,’ tannins must be highly bound, and possibly precipitated in order to prevent tannin-non-heme iron chelation [47, 51]. As PRP profiles change due to tannin consumption, favoring bPRPs and gPRPs [4] that may bind a greater number of tannins before crosslinking leads to complex formation, molecules that randomly dissociate may chelate non-heme iron in digestion. This may be more commonly found in tannins of lower affinity (lower polymerization, galloylation, or hydroxylation).

#### In vivo findings

In vivo, it is interesting to note that overall PRP production closely follows non-heme iron absorption (Fig. 5). Protective benefits of PRP production against tannins have been particularly highlighted in hamsters, which do not produce PRPs [31]. Other studies have shown that PRPs play a role in acclimation to tannins [36, 83–85], although this is not always without reductions in growth overall compared to control [30]. Non-heme iron absorption is generally impacted by tannin intake, although major limitations, including interaction of tannins and non-heme iron in solution before exposure to PRPs [37], reductions in overall chronic non-heme iron absorption [70], and differences in measurement of PRPs between both studies limits the generalizability of these findings. It is particularly important to point out that non-heme iron absorption was highly variable in all experiments presented, highlighting the importance of individual variability in non-heme iron absorption and the need to understand mechanisms behind non-heme iron inhibitors and enhancers from a physiological point of view. Interestingly, non-heme iron absorption and non-heme iron status (measured by hepatic non-heme iron) were not correlated in these studies, possibly indicating that either non-heme iron supplied was greater than the burden of tannin supplied, or that numerical non-heme iron absorption may be a poor indicator of non-heme iron status (in this limited model). Adequate hepatic non-heme iron stores likely impacted non-heme iron absorption percentage changes when comparing acute to chronic tannin challenges [70], although there was an increase in non-heme iron absorption suggesting that there was accommodation to poor non-heme iron absorption in acute challenges [37]. Perhaps more interestingly, rats gavaged with tannins had similar non-heme iron absorption and hepatic status compared to those with oral tannin exposure, suggesting that mucosal or endocrinological protection mechanisms are additionally important to non-heme iron bioavailability with tannin consumption. It may be that immediate tannin accommodation starts with PRPs, but is more efficiently dealt with elsewhere.

## Conclusion

Similarities in binding mechanisms to tannins may support the hypothesis that PRPs play a role in protecting against tannin-non-heme iron chelation during digestion. Additionally, PRP production is linked to non-heme iron absorption, although absorption is poorly correlated with non-heme iron status in reviewed studies. More research is needed to explore changes in PRP production in humans related to tannin-non-heme iron chelation, and in vitro studies specifically modeling tannin-non-heme iron chelation in the presence of food tannins. Further in vivo research should explore the differences between condensed and hydrolyzable tannins on non-heme iron status, and explore potentiating effects of antinutritional factors when consumed together.

## Additional file

Additional file 1: A) Quality assesssment of studies, B) Biochemical study characteristics, C) Inculsion and exclusion criteria. (DOCX 27 kb)

## Abbreviations

aPRP: Acidic proline-rich proteins
bPRP: Basic proline-rich proteins
gPRP: Glycosylated proline-rich proteins
PICO: Population intervention comparison and outcomes
PRPs: Salivary Proline-Rich Proteins
