# Supplementary material for: Salivary proline-rich protein may reduce tannin-iron chelation: a systematic narrative review
Source: Nutr Metab (Lond). 2017 Jul 24;14:47. doi: 10.1186/s12986-017-0197-z (PMC5525358; doi:10.1186/s12986-017-0197-z)
Supplement: Additional file 1: — A) Quality assesssment of studies, B) Biochemical study characteristics, C) Inculsion and exclusion criteria. (DOCX 27 kb) [file 12986_2017_197_MOESM1_ESM.docx]

Appendix A: Quality assessment of studies:

(Each criterion assessed as having high, unclear, or low risk)

1. Is there a clear purpose for study aims and research?
2. Is there relevant background information presented?
3. Methods assessment:
   1. Are methods employed appropriate?
   2. Were methods completely described or referenced?
      1. Studies described methods for allocation of samples or groups to treatments, if applicable
         1. Appropriate sample size or sample size justification
      2. Are instruments used for data collection appropriate for outcomes assessed?
      3. Methods allowed for randomization of study groups to minimize outcomes bias, if applicable
   3. Was the intervention independent of other changes (minimization or accounting of confounding variables)?
4. Outcomes measurement
   1. Were all outcomes measured reported?
   2. Completeness of outcome data:
      1. Are data presented clearly related to methodological design?
      2. Did studies, if applicable, account for attrition or negative outcomes to intervention or control groups?
      3. Is analysis of data reported, and appropriate for study design?
      4. Are data transformations accounted for?
5. Conclusions:
   1. Do conclusions and discussion match findings presented?
   2. Do findings contribute to current theory or future practice research?

Appendix B:

Biochemical study Characteristics

| Study | Topic | Tannin-non-heme iron binding (x) or tannin-PRP binding (o) | Tannic acid - Proanthocyanidin comparison | Tannins used | Size of tannins compared | Measurement of mechanism | Conditions for assay | Findings |
| --- | --- | --- | --- | --- | --- | --- | --- | --- |
| ^(77)^ | Effect of AA or EDTA on polyphenol non-heme iron binding | x | x | tannic acid, gallic acid, catechin, coffee, tea | monomers | catechol/galloyl equivalent measurement of non-heme iron binding capacity | | 900 µg/ml polyphenol bound less non-heme iron than at 40 µg/ml (<50% vs. 100%); tannic acid more predilection to non-heme iron binding ability vs. gallic acid or catechin |
| ^(51)^ | IB-8c (basic PRP) and GSE interactions influenced by polysaccharides in wine | o |  | proanthocyanidins | procyanidin tetramers, pentamers, gallate | dynamic light scattering (amount of precipitation) | 31.2 mg/L tannin: 0.6-5mg/L IB8c and 3.12mg/L IB8c: 19.5-46.8 mg/L tannin; pH 5.0, 12% ethanol, 20C | 20.8 uM needed for precipitation of PRPs. Plateau of PRP binding at 3.12 mg/L (tannin/IB8c molar ratio is 35); this means that at a given concentration of tannin, PRP are bound to a maxima (multiple PRP per tannin) and then decrease at a certain concentration- diffuse). At a higher tannin to protein ratio, there is also a plateau of precipitation. At increasing ionic concentrations, precipitation increases; explained by increasing hydrophobicity (polyphenol rings stack) |
| ^(54)^ | Affinity of GSE twoard PRP in whole saliva | o |  | proanthocyanidins | dimers, trimer,tetramer (average polymerization 3.2; mW 936) | HPLC, SDS-PAGE, tryptic digestion, Mass spec and top down analysis/MALDI-TOF | pH 5.0, 20C, 12% ethanol/saliva (0.00-1.5 mM GSE in saliva) | histatin, statherin, aPRP first bind, then bPRP and gPRP at higher concentrations (min @ 1.25) |
| ^(55)^ | binding affinity of different PRP to hydrolyzable vs. condensed tannins | o | x | Quebracho tannin vs. tannic acid | not noted | competitive binding assay at different concentrations of tannin | pH 7.4, 10 µg protein and tannin, 37C. Stability testing in HCL with or without pepsin for 210 minutes; 7.4 c/trypsin/chymotrypsin/carboxypeptidase | Plateau of precipitation at2- 3 µg of condensed tannin/tannic acid: more bound condensed tannin vs. tannic acid (40 µg /assay vs. 30 µg /assay. No glycosylated binding of condensed PRP, some of tannic acid, most binding to BPRP. At pH 2.0, 93% of complexes were insoluble (condensed tannin) vs. 71% hydrolyzable; small intestine c enzymes 72% insoluble vs. 53% hydrolyzable |
| ^(67)^ | comparison of bioavailability of tannins with and without PRP | o |  | tannic acid | tri, tetra, pentagalloyl glucose | caco-2 cell transport with/without PRP presence | 5-90 uM 5GG concentration | Increased concentration of 5GG decreased absorption, addition of Ib4 to fraction decreased absorption 8-9 fold (90uM 5GG). Precipitation of 5GG 4.5:10 5GG:IB4 |
| ^(56)^ | concentration effects on binding; stereochemistry effects | o | x | epigallocatechin, gallic acid, 5GG | pentagalloyl glucose, monomers | tannin protein binding competition assay | PRP isolated from whole saliva as 0.1 µg/ml solution of saliva or with buffer (pH 9.6) with varying concentrations for 5-20 min. | higher proline content to bPRP and gPRP (0.11-1.22 µM displacement constants- which means that there was not much required to bind; vs. 0.54 um lowest in bacon and Rhodes 1998; in proanthocyanidin monomers to 350 µM for highest- hydrolyzable are low); galloylation decreases displacement constants |
| ^(46)^ | dimer (B2) vs. pentagalloylglucose vs. trigalloylglucose vs. proanthocyanidin monomer vs. epicatechin vs. propyl gallate | o | x | see topic | see topic | NMR | 40 mM B2, 50 mM PGG/epicatechin: 0.5ml 4mM PRP or 2mM PRP pH 3.8 | N terminal proline shifts this is calculated by chemical shifts (binding site for tannin); the hydrolyzable tannins had H bonding (protons in tannins bound to protons of PRPs); not true of condensed tannins (non-heme iron binding is protonated); Ka B2 = 300/M vs. PGG 241, TGG 28, epicatechin 39; increased ring structure and size leads to cross linking of polyphenols bound to PRP; does not occur in hydrolyzable tannins= also, larger size of polyphenol reduces N terminal sites that can bind on PRP but increase hydrophobic interactions (increasing precipitation), and the proline residues at the N terminal then functions to not only bind galloyl group, but to allow for secondary interactions with other galloly functions. Predominant force for binding is hydrophobic; essential polyphenol site for binding is a proline residue with an amide bond and amino acid residue; polyphenols form multidentate bonds: the TGG PGG B2 polyphenol curves showed that there is cooperative binding between aromatic functions on polyphenols and the neighboring polyphenol sites that are on the PRP peptides; noted that polyphenols start to self-associate when bound: larger compounds more than smaller |
| ^(47)^ | B1, B3, C2 procyanidins and PRPs to look at the process of binding | o |  | B1/B2 dimers, C2 trimer | B1/B2 dimers, C2 trimer | NMR | pH 3.5; 0.5-20mM PRP (exp 1) 15.7mM tannin, 1.7mM protein | At below tannin concentration 10 mM the interaction is specific, then second phase at 10 mM there is a linear chemical shift with tannin addition (non-specific interaction where tannins exist in aggregates); longer proteins are better, due to wrapping of the PRP around the tannin. Again, Kd C2 > B1 > B3. PRP IB9 binds to 2-3 tannins each |
| ^(68)^ | types of tannins and the quality of binding with PRPs | o | x | proanthocyanidins | dimers (B1-9) and trimer C1, B2 3-O gallate, epicatechin from GSE | nephelometry (stereochemistry)- tannin specific activities (TSA), nephlos turbidity units (NTU) | 12% ETOH, pH 5.0 or 3.5; 760 µg procyanidins, 48 µg PRP (15:1) | Stabilization of precipitates at 40 minutes; stereo specificity mattered for binding: c6 vs. c8 binding; gallic acid esterification (Table 1: TSA) explored. NOTED THAT SMALL POLYPHENOLS BIND BUT DO NOT CROSSLINK; THAT THE PYRANIC RING STRUCTURE OF FLAVAN 3-OL INCREASES ABILITY TO INTERACT WITH PROTEINS. BIGGER POLYMERS BIND WITH MORE AFFINITY. ALSO, C8 BONDS > AFFINITY THAN C6 BONDS. |
| ^(48)^ | understanding effect of gallolylation, polymerization, and B ring hydroxylation on PRP binding | o | x | EgC, ECG, B2, B2 3-O gallate | dimers, monomers | ESI-MS | protein polyphenol 1:10, 24C, pH 3.2 | Galloylation favors binding. Binding is specific to tannins (did not bind to compound similar in structure); binding in phases: 1) dissociation is minimal 2) log-linear dissociation 3) plateau (how strong is the bond): dimers > monomers; OH group on the B ring = improved stability. Importance of features: B ring hydroxylated < galloylation < degree of polymerization; procyanidins are mostly composed of epicatechin and catechin units |
| ^(57)^ | BPRP with various wavelengths against polyphenols | o |  | flavonoids, oligomeric flavan-3-ols | flavonoids, oligomeric flavan-3-ols | ESI-MS | 1:1 peptide polyphenol conditions (gas) 3:1 peptide: polyphenol in 10% ETOH for 1 hour | C2 > B2 > quercetin > B1 > B4 > B3 > epicatechin > catechin; larger molecules display greater affinity; stereochemistry at C3 makes a difference (epicatechin (2R, 3R)/catechin (2R, 3S); dimers > monomers; C2 stereochemistry matters (2R > 2S); OH groups on the B cycle > not hydroxylated; B cycle branching position (B cycle bound to 2C of flavonoid skeleton greatest); increased length of the PRP peptide increases binding; they hypothesized that OH groups and stereochemistry of the OH groups promoted H bonding, increasing interaction with PRPs |
| ^(65)^ | influence of gastric digestion on PRP tannin complexes | o |  | GSE | catechins, gallic acid, procyanidin dimers, trimers, tetramers, pentamers, hexamers/ galloyl derivatives | HPLC, in vitro digestion | saliva: wine ratio of 2:1 (well justified) pH 3.5; pepsin c pH 1.7 for 2 h | Tannin/SP complexes that are monomers, dimers, trimers are disrupted by gastric digestion. Tetramers, pentamers are more resistant; statherin, aPRP bind more potently, then gPRP, then bPRP |
| ^(49)^ | influence of EgCG on IB5 | o |  | EgCG | EgCG | MS/ SAXS, DLS | pH 5.5 ; protein = .336mM (1-3.5 mg/ml), stimulated to mimic saliva | Threshold concentration of tannins, below this, the DLS intensities were the same as the pure protein solution; at threshold, there was an almost exponential increase in precipitation with increased concentration. At a higher concentration, it took much more EgCG to precipitate proteins; PRP can bind 1-8 tannins depending on the concentration, at higher concentration of tannin, 1 PRP can bind up to 14 tannin molecules meaning that the 'n' binding sites does not correlate to tannin binding at higher tannin concentrations because the tannin 'stacks'. E50 K 1-8 are similarly stable, greater than this is a different interaction (tannin stacking- hypothesized by authors); binding sites are independent and have free energy; found that even when not precipitated, PRP were binding a significant amount of tannin (up to 3 per molecule- could be why precipitate analysis does not show bPRP although these seem to bind more potently in other experiments- not hitting threshold); at precipitation, there is aggregation that may be attributed to the multidendate nature of tannins (crosslinking) that conserves PRP measures; the limitation in aggregation continuing are 'poison' PRP that do not carry enough tannins to continue aggregation (cannot crosslink); tannins move fairly easily on the PRP polypeptide chain, and thus will dissociate easily when population has not been met; in fact, at low ionic concentrations, tannins and PRP repel each other |
| ^(61)^ | influence of pH on tannin salivary protein binding | o | x | hydrolyzable and condensed extracts, catechin, tartaric acid, gallic acid | mixed in extracts, not specified | diffusion precipitation interaction assay | pH 3.5 vs. pH 7.0 | pH 3.5 precipitates tannins > pH 7.0 |
| ^(53)^ | influence of tannin binding on PRP conformation | o |  | EgCG | EgCG | mass spectrometry coupled with ion mobility | IB5 and 12% ETOH, pH 3.5; molar ratio of 1:20 IB5:EgCG | Poisson binding (continuous and independent binding in favor of 'non cooperative sticking process') binding 1-9 tannins; at binding of 1-7 tannins, IB5 structure is stable (in its still unstructured state) at 10 ligands, a more compact conformation is formed that is smaller than the unstructured protein; several tannins are required to stabilize the folded state. Folding is favored by H bonds between PRP and tannin OH groups on tannins that stack together |
| ^(66)^ | influence of polymerization on precipitation (as a surrogate of affinity) | o |  | GSE | monomers, dimers, trimers | HPLC DAD | 37C incubation, 1,2,8 ml of saliva mixed with 40 ml GSE or 20/40 ml GSE sipped | increased tannin concentration increased precipitation; of LMW tannins, ECG precipitates effectively; at low-EGCG -protein ratios, binding is progressive in suspension without binding, at higher ratios, precipitates |
| ^(4)^ | glycosylation and influence on tannin binding | o |  | GSE | monomers and galloylated units | SDS-PAGE | 10% ETOH, 20 µg purified protein or 40 µl saliva, different concentrations of tannins | 20.57% of GSE had galloylated units; compared astringency trained and untrained individuals: gPRP more prevalent in trained saliva; 30% of tannins were precipitated by the astringency trained individual (most remained in supernatant), 85% in the astringency untrained (all precipitated); precipitate was dose dependent and selective; the 'nature of the saliva' significantly predicted the precipitation of tannins as well; polymerization of tannins positively correlated with precipitation; however, trained saliva was able to precipitate smaller polymers than the untrained despite similar AMOUNTs of protein in saliva; bPRP most commonly precipitated and the trained participant had higher quantity than the untrained; there were no proteins in the untrained supernatant; there were abundant glycosylated PRP in the trained supernatant. PRP themselves (purified) are more effective at binding tannins than whole saliva however, PRP affinity greater than alpha amylase. At low tannin concentration, all polymers were bound, at higher concentration, selective to higher order polymers. Glycosylation seems to stabilize the complex to tannin outside of precipitation. At low tannin concentration, glycosylation may stay soluble by decreased hydrophobicity, while at higher concentrations, hydrophobicity is reduced, and the complex precipitates. |
| ^(42)^ | interactions of IB5 with EGCG | o |  | EgCG | EgCG | DLS, isothermal titration microcalorimetry, dichroism | pH 3.5 ionic strength at 100 mM (same as saliva mixed with wine) 6.4 or 12.8 mM EgCG solution with 0.25-2 mg/ml IB5 | PRP are random and unfolded; At low protein concentration, no aggregates occurred until a high tannin ratio then would slowly start. At a next saturation, there was a rapid increase in aggregation and further increases in tannin led to immediate precipitation. If the solution was started at very high tannin concentration, immediate aggregation and precipitation. At a lower tannin ratio (more protein), the precipitation occurred at a lower threshold. 3 stages at low concentration: 1) saturation of protein residues with tannin 2) bridging of the proteins by tannin and hydrophobic stacking of tannins 3) aggregation and phase separation. Protein folds around the tannin and then later tannin bridges the condensed protein aggregates. |
| ^(58)^ | influence of tannin stereochemistry on PRP binding | o |  | GSE | monomer free; mostly catechin, epicatechin, epicatechin 3-O-gallate | ITC | 10% ETOH, 40 µl saliva and different amounts of tannins | Individual salivary profiles differed greatly throughout the day and by individual; PRP came out of supernatant as tannin concentration increased. Glycosylation increased threshold for precipitation but did not impede affinity. Larger proanthyocyanidins precipitate first, LMWP stayed in supernatant longer. |
| ^(59)^ | influence of glycoprotein on binding | o |  | sorghum and Quebracho | multiple polymers of PACs |  | pH 4.8, methanol, acetate buffer | < binding to sorghum than Quebracho, deglycosylated proteins with less affinity suggesting that is important; glycosylation increased solubility (increased tannin binding in solution) |
| ^(62)^ | influence of pH, fructose, ethanol on precipitation of salivary proteins | x | x | tannic acid or wine | multiple polymers of PACs | SDS PAGE | 25C with either tannic acid or wine (pH 3.6/3.6) with 2-4-6 g/L fructose, manoproteins, tartaric acid. 11% ETOH | Lower pH increased binding > decreased hydrophobicity and increased hydrophobic binding; this is more prominent in wine than in tannic acid (increase in 21.7 vs. 11, 9 respectively). ETOH/fructose negatively effects precipitation (disruption of H bonds between polyphenols and proteins; ETOH may change conformation of PRP and solubility of tannin and change reactivity; carbohydrate molecule CH2OH may also reduce binding sites to proteins by binding to NH3 proline surfaces on proteins; as pH decreases, wine more commonly precipitates before tannic acid |
| ^(50)^ | PRP alleviation of intestinal enzyme binding by tannins | o |  | EgCG | EgCG | in vitro digestion stability, absorbance for reactivity, SDS PAGE, HPLC | gastric juice pH 2.07 c pepsin; duodenal juice pH 7.8 c/ lipase, pancreatin | with PRP: IC50 of chymotrypsin, trypsin, lactase increased 2-3x; maximum pH effect of PRP at ~5-6, lower had increased protein binding, higher reduced (Figure 3); PRP bound and recovered EgCG >2x in duodenal digestion, no effect in gastric or control solutions; PRP are resistant to proteolytic digestion |
| ^(60)^ | saliva and hydrophobic interactions with tannins | o | x | wine tannins | multiple polymers of PACs | TRAP | 1:5 saliva to wine ratio with acidic extraction (1% TFA) | Moderate removal of polyphenol with 1 ml saliva |
| ^(45)^ | binding mechanism tannins | o | x | EGCG, EGC, PGG | penta and tetra gallotannins | NMR, diffusion, dynamic light scattering | 20mM polyphenol solutions 2mM PRP (mouse PRP); pH 3.8 | Preferential binding to polyphenols at the proline residues; but interactions also occur at the arginine and phenylalanine side chains; polypeptides self-associate and so the number of binding sites does not equal the concentration of solution. The strongest self-association is PGG; ECG and EGCG same self-association suggesting that H bonding is not primary mode of self-association, but hydrophobic bonds are. Most binding sites on PRP can be filled simultaneously; arginine strengthens the bonds of tannin-phenylalanine/proline, but does not independently bind. Longer PRP 'wrap' around tannins (higher affinity); at lower temp, multidendate binding. Binding affinity: PGG>TGG>TG>EGCG~EGG. Stages of binding: reversible hydrophobic binding of polyphenol that gives a soluble complex due to stacking, particularly of hydrophobic galloyl rings. 2 addition of polyphenol, and two peptides are crosslinked by two polyphenol protein interactions, and the complex becomes insoluble; this is different for different polyphenols, for example: 3:1 EGCG vs. 0.5:1 PGG 3) phase separation and aggregation of insoluble complexes determined by the surface charge (EGCG: pH 3.8; 50% monomers, 2.3/3 binding sites occupied (monomer and dimer binding) vs. PGG 64% monomeric, 0.4/3 binding sites are occupied at precipitation. |
| ^(52)^ | type of polyphenol tannin binding | o |  | catechin, epicatechin, B2, C1 | dimers, trimers, monomers | RPHPLC | pH 3.2 either 1:1 or 1:2 v/v mixture; 37C 90 min incubation | Flavone type and concentration of protein effect precipitation, but there is also a significant interaction between the compound and saliva; not all flavones behave the same way in saliva; C1 < epicatechin < catechin < B2; protein concentration> = more precipitation. Increased concentration of polyphenol = more precipitation. More protein had pronounced effect on polyphenols with less affinity (C1 and epicatechin); most pronounced reduction in affinity with smaller molecules. In lower concentrations of monomers, increased precipitation of protein |
| ^(64)^ | interaction of PRP with galloyl ring and pyrrolidine ring | x |  | tannic acid | monomers, dimers, trimers | NMR | 1:0-1:5.6 ratio of PRP to polyphenol | Increase in temp or decrease in pH solubilize complexes. Changes in chemical shifts suggested binding rather than conformational changes; proline is key binding site. More complexes bound = less dissociation. |
| ^(63)^ | stereochemistry of tannins | o |  | EgCG | EgCG | NMR | pH 3.5; 100 mM and 5 mM NaCl | Ionic concentration changes conformations of PRP to be more structured at 100 mM, although both have unfolded conformation and there is a higher level of disorder in the PRP at 100 mM. On binding to EGCG, secondary structure of the protein was changed at 100mM but not 5 mM; NMR modeling suggests beta sheet structure of Proline residues after tannin binding; residual sites are preferential for binding |
| ^(43)^ | mechanism of binding | o |  | EgCG, B2, B23OG | dimers, EgCG | MS-SAXS | Interaction of IB5 with various tannins; water/ETOH 88:12 at pH 3.3. 10 µM IB5 to tannin solution for ratio of 1:10 protein: polyphenol | Polyproline helix and repeat proline sequences are most preferentially bound, they are surrounded by glycine and alanine which give more flexibility and establish increased number of H bonds. While PRP are unstructured, the polyproline regions are rigid and provide anchoring points to tannins. Shorter PRPs do not change conformation around tannins. Noted that extended tannin structure also precipitated binding |
| ^(44)^ | mechanism of binding | o |  | B3 | Trimer | MS-circular dichroism | pH 3.5 | tannin binding does not modify peptide folding; at low concentration, 34% TII helix, 66% extended and random colloid conformation; there are extended conformations with a type II helix with 7 residues (IB7); initial H bonding to proline residues; the PRP studied was amphiphilic, tannin binding to the hydrophilic face (H bonds) |

Appendix C:

Inclusion criteria:

1. Studies that include long-term effects of tannin consumption on PRP outcomes and non-heme iron bioavailability
2. Studies that include at least two key words, tannin AND salivary proline-rich protein OR non-heme iron

AND

3.) Studies that explore biochemical modeling for binding mechanisms of tannins and PRPs

4.) Studies that explore binding affinity for PRP and tannins

5.) Studies that compare mechanism of non-heme iron-tannin chelation to Tannin-PRP binding

Exclusion criteria:

1. Validity studies for measurement tools
2. Assessment studies of food tannin content or food non-heme iron content
3. Studies that include surrogate PRP proteins (albumin, BSA, amylase, gelatin)
4. Studies that explore PRP without tannin or non-heme iron binding
5. Ruminant modeling
6. Specialized conditions: cancer, Sjogren’s syndrome, burning mouth syndrome, hemochromatosis
7. Studies did not present comparative findings (descriptive or qualitative studies describing astringency or optical tongue without biochemical mechanisms/affinity studies)
8. *In vivo* animal studies that did not explore non-heme iron bioavailability with PRP and tannins
